# Traditional medicines as a mechanism for driving research innovation in Africa

**DOI:** 10.1186/1475-2875-10-S1-S9

**Published:** 2011-03-15

**Authors:** Ivan Addae-Mensah, Foluke Fakorede, Andreas Holtel, Solomon Nwaka

**Affiliations:** 1 Department of Chemistry, University of Ghana, Legon, Ghana; 2 UNICEF/UNDP/World Bank/WHO Special Programme for Research and Training in Tropical Diseases (TDR), World Health Organization, Geneva, Switzerland; 3 European Commission, DG Research - Health Research Unit, Brussels, Belgium

## Abstract

The outcomes from recent high profile deliberations concerning African health research and economic development all point towards the need for a mechanism to support health innovation on the continent. The mission of the African Network for Drugs and Diagnostics Innovation (ANDI), is to promote and sustain African-led health product innovation to address African public health needs through the assembly of research networks, and building of capacity to support human and economic development. ANDI is widely viewed as the vehicle to implementing some of these recommendations. There is tremendous opportunity for Africa, to leverage the expertise in natural products and traditional medicines in support of this objective to kick-start innovation. This report highlights key recommendations that have emerged through expert forums convened by ANDI on the challenges, opportunities and prospects for investing in this important area of research.

## Background

The African Network for Drugs and Diagnostics Innovation (ANDI)[1] was conceived and launched as a concept at Abuja in 2008 by the World Health Organization through TDR and its African and East Mediterranean Regional Offices; several African institutions, policy makers, Africans in the Diaspora, the African Development Bank (AfDB) and other stakeholders. As a major international partner, the European Union has also provided support, both through the EU Commission's EuropeAid Cooperation Office and through the EUs' 7^th^ Framework Programme for research (FP7). Progress in the past year on the establishment of ANDI has led to the identification and selection of the United Nations Economic Commission for Africa (UNECA) as the host agency for ANDI in Africa[2]. ANDI intends to develop and fund a portfolio of collaborative network projects and leverage existing capacity in Africa in a manner that will support capacity and infrastructural development for product research, development and delivery. The key objective is to discover, develop and deliver affordable new drugs, vaccines, diagnostics and other health products (including those based upon natural products and traditional medicines) to fight and prevent diseases that disproportionately affect the African continent.

Over the past year, ANDI has convened its stakeholders to discuss and debate mechanisms to advance R&D, including those based on traditional medicines and natural products, for diseases that afflict Africa, and how these can be prioritized and implemented. This report provides an overview of those discussions and the resulting recommendations. It is important to note that although the disease focus of this special issue of the *Malaria Journal* on natural products is clearly malaria, the issue of traditional medicines is far more cross-cutting and goes beyond disease and/or specific research area.

## Support for Traditional Medicine R&D in Africa

### The MIM symposium

The 5th Multilateral Initiative for Malaria (MIM) Conference in Nairobi, Kenya (November 2-6, 2009), attracted approximately 2,000 international delegates covering the wide scope of research on malaria from malaria control, treatment and efforts towards eradication, vector control strategies, drug discovery & development activities and vaccine discovery. During MIM, a joint symposium between the European Union (EU) and WHO/TDR was convened to define future research and development needs in the field of drugs and diagnostics as part of the work on ANDI. It also aimed at elaborating how EU's 7^th^ framework programme (or successive programmes) might usefully contribute towards strengthening research and development efforts to eliminate malaria in the long term. Although the focus of the MIM meeting was malaria, the product R& D and access challenges and possible solutions discussed, in many ways cut across diseases and are applicable to R&D activities in developing countries, irrespective of the region. The symposium attracted more than 100 participants that included African and non-African researchers, funding and research network organizations. A selection of varied presentations covering drugs, diagnostics as well as natural products - based discovery, development and delivery placed the current situation in context and initiated a very lively debate and discussions. The resulting consensus document highlighted a number of priority research areas. Excerpts from the report of relevance to the topics of this special issue are:

#### Novel chemical entities

The paucity of novel chemical series to enter the development pipeline for malaria and the neglected diseases [3], was highlighted throughout the MIM conference. This is indicative of the limited approaches and support for preclinical drug discovery efforts. The need for increased access to a broader collection of validated drug or diagnostic targets; the exploration of broader chemical space either through the assessment of natural products, traditional medicines or synthesis of small molecules; and repurposing of known drugs for other indications were stressed at the meeting. A major issue highlighted by several African scientists was how to better advance natural products and traditional medicines as a source of new drug leads and formulation. In this context, it was agreed that the field of phytochemistry required further development to support the discovery of new chemical entities rather than just focusing on publications as the final output. The need to encourage and support well-coordinated multidisciplinary partnerships/networks to leverage available expertise was agreed as the way forward. It was also agreed that a critical mass of medicinal chemists, pharmacologists and toxicologists were needed in Africa and strengthening these areas would require more investment and coordinated R&D in Africa through ANDI and other initiatives. Clinical evaluation and registration of new products could be pursued in collaboration with complimentary programs such as the European Developing Countries Clinical Trial Partnership [4].

#### Traditional medicines

Could the next major breakthrough in malaria chemotherapy come from natural products and traditional medicines?, Will Africa be the source of such a breakthrough? African researchers believe that Africa has a lot to offer from its vast biodiversity resources that could in effect provide answers to these questions, if appropriate measures are taken. It is known from work in Asia and South America that this indeed possible.

It is known that up to 80% of African populations use traditional medicines in order to address their health issues [5]. In spite of this, the biodiversity of Africa has yielded few examples of standardized phytomedicines and only limited systematic analysis of available medicinal plants has been undertaken. Although some pharmaceutical products such as Niprisan? (for sickle cell anaemia) are commercially available based on work done in Africa [6], more work is required to develop a standard process for the evaluation and regulation of traditional medicines or any resultant products. Many argue that this evaluation requires modern product R&D processes such as biology, computational methods, medicinal chemistry, DMPK as well as toxicology, such that it should be possible to develop standardized phytomedicines as it is done for drugs based on synthetic compounds. A common argument is that the optimal efficacy of these plant-based products is inherently linked to the mixture of efficacious chemical entities they contain and hence they should be developed as both herbal formulations and also form the basis of medicinal chemistry programmes. Concerted efforts should be made to clearly define processes and guidelines for traditional medicines and natural products research, in order to support the advancement of this important field. The development of traditional medicines should not be seen as the answer to providing cheap treatments to poor populations. It is important that such medicines are given the same scrutiny as any other pharmaceutical to ensure protection of patients from any major adverse event and also to ensure the benefits of optimal efficacy. Research in support of the use of traditional medicines and natural products in disease prevention and control should also receive attention, for example, vector control. In all cases, consideration must be given to the sustainable use of medicinal plants/natural products and the conservation of the habitat in which they occur.

The workshop also identified enhanced efforts for Health Systems research, and more R&D on diagnostics, vaccines and enabling technologies as further priority research areas for the African continent. All should be tailored to support African priorities, needs and encourage research leadership.

## The 3^rd^ ANDI Stakeholders Meeting

The recently concluded 3^rd^ ANDI Stakeholders Meeting (11-13 October 2010, United Nations Office in Nairobi, Kenya) provided a forum for over 500 stakeholders from 40 countries to discuss progress made towards the implementation of the ANDI strategic business plan which is based upon findings from a situational analysis [7,8]. Amongst other things, this key document outlines plans to develop a portfolio of high quality, pan-African health product R&D projects through direct funding and project management; provide support for a network of research centres of excellence as well as develop an IT platform for knowledge management in Africa. ANDI will also broker relationships with key stakeholders and funders through advocacy and the fostering of project partnerships between the public and private sectors.

Highlights of the Nairobi meeting included the inauguration of the ANDI Board, launch of the first call for projects and the launch of the ANDI R&D and Knowledge Management Databases. During the meeting a round-table session on traditional medicines brought together several experts who provided their perspectives with further insight from the audience. The goal of the discussion was to specifically address the opportunities and issues related to Traditional Medicines (TM) including plant-derived anti-malarials. These include:

• The need to better define the processes for R&D on Traditional Medicine (TM), including validating processes to ensure safety, efficacy and efficiency and developing a scientific rationale for each product.

• Need for policy and guidelines in the development of TM products in Africa

• Ethical and regulatory issues on TM

• Establishment of a holistic intellectual property management policy that will address the intellectual property issues arising from the use, development and commercialization of traditional medicines.

• Bridging the gap between TM practitioners and scientists.

• The role of TM in achieving MDGs in Africa.

These all point to the urgent need for a framework to provide the necessary support in addressing these needs. The report of the round table is being finalized for publication in due in course.

## Defining a framework to address these needs

In summary, the consensus was that the African research community requires a mechanism for translating the knowledge emerging from their research programmes into products. It is important that this mechanism incorporates capacity building based on tangible projects and local needs.

An important outcome from these discussions was the elaboration on the context and framework that will allow cooperation of various partners and donors to better interact and support research efforts in Africa. Certainly, a more product-orientated research approach led by Africans is needed. ANDI is now increasingly highlighted as a unique opportunity to help advance this approach, in collaboration with Northern and other Southern partners as well as Product Development Partnerships (PDPs) such as Medicines for Malaria Venture (MMV), the Drugs for Neglected Diseases Initiative (DNDi), the Foundation for New Innovative Diagnostics (FIND) and the Global Alliance for TB Drug Development (TB Alliance). Opportunities for synergy could also be found in several European and American funded R&D cooperative programmes. An example is the EU ANTIMAL (for research into anti-malarial drugs), which offers a valuable framework for product-orientated drug discovery research undertaken by a number of African research groups alongside European malaria drugs researchers. This EU-funded drugs research effort focuses on more upstream discovery research, which in future initiatives could be complemented by the industry-type product development promoted through partnerships, for example, PDPs or regional innovation networks like ANDI.

There has certainly been a renewed global interest in TM and natural products research over the past few years from all quarters (academics, industry, TM practitioners and policy makers). Whether the use of traditional and modern medicine should be harmonized in Africa to ensure the development of safe, effective and affordable products, and whether the development of TM-based products should follow the classic western R&D paradigm is a debate that will continue. However, all are in agreement that the time has come for African researchers and policy makers to capitalize on an area that is widely viewed as the continent's "competitive advantage". By doing so (and ensuring that this is done in a manner that is sustainable), will hopefully provide the much needed boost to innovation, economic and health development on the continent.

## Competing interests

The authors declare that they have no competing interests.

## Authors’ contribution

All authors contributed in the conception and design of the report; FF and SN drafted the manuscript; IAM and AH critically reviewed and revised the manuscript. All authors read and approved the final manuscript.
